# Prognostic Value of Soluble AXL in Serum from Heart Failure Patients with Preserved and Reduced Left Ventricular Ejection Fraction

**DOI:** 10.3390/jpm13030446

**Published:** 2023-02-28

**Authors:** Helena Cristóbal, Cristina Enjuanes, Montserrat Batlle, Marta Tajes, Begoña Campos, Josep Francesch, Pedro Moliner, Marta Farrero, Rut Andrea, José Tomás Ortiz-Pérez, Albert Morales, Manel Sabaté, Josep Comin-Colet, Pablo García de Frutos

**Affiliations:** 1 Department of Cell Death and Proliferation, Institute of Biomedical Research of Barcelona (IIBB-CSIC), August Pi i Sunyer Biomedical Research Institute (IDIBAPS), E08036 Barcelona, Spain; 2 Community Heart Failure Program, Department of Cardiology, Bellvitge University Hospital, E08907 L’Hospitalet de Llobregat, Spain; 3 Bio-Heart Cardiovascular Diseases Research Group, Bellvitge Biomedical Research Institute (IDIBELL), E08907 L’Hospitalet de Llobregat, Spain; 4 Centro de Investigación Biomédica en Red de Enfermedades Cardiovasculares (CIBERCV), E28029 Madrid, Spain; 5 Cardiology Department, Clinical Cardiovascular Institute, Hospital Clinic and IDIBAPS, University of Barcelona, E08036 Barcelona, Spain; 6 Department of Basic Clinical Practice, Universitat de Barcelona, E08036 Barcelona, Spain; 7 Department of Clinical Sciences, School of Medicine, University of Barcelona, E08036 Barcelona, Spain; 8 Hospital del Mar Medical Research Institute (IMIM) and IIBB-CSIC Associated RDI Unit, E08036 Barcelona, Spain

**Keywords:** heart failure, biomarker, receptor tyrosine kinase, sAXL, prognosis, cardiovascular disease, preserved ejection fraction

## Abstract

Heart failure (HF) is classified according to the degree of reduction in left ventricular ejection fraction (EF) in HF with reduced, mildly reduced, and preserved EF. Biomarkers could behave differently depending on EF type. Here, we analyze the soluble form of the AXL receptor tyrosine kinase (sAXL) in HF patients with reduced and preserved EF. Two groups of HF patients with reduced (HFrEF; n = 134) and preserved ejection fraction (HFpEF; n = 134) were included in this prospective observational study, with measurements of candidate biomarkers and functional, clinical, and echocardiographic variables. A Cox regression model was used to determine predictors for clinical events: cardiovascular mortality and all-cause mortality. sAXL circulating values predicted outcome in HF: for a 1.0 ng/mL increase in serum sAXL, the mortality hazard ratio (HR) was 1.019 for HFrEF (95% CI 1.000 to 1.038) and 1.032 for HFpEF (95% CI 1.013 to 1.052). In a multivariable Cox regression analysis, sAXL and NT-proBNP were independent markers for all-cause and cardiovascular mortality in HFpEF. In contrast, only NT-proBNP remained significant in the HFrEF group. When analyzing the event-free survival at a mean follow-up of 3.6 years, HFrEF and HFpEF patients in the higher quartile of sAXL had a reduced survival time. Interestingly, sAXL is a reliable predictor for all-cause and cardiovascular mortality only in the HFpEF cohort. The results suggest an important role for AXL in HFpEF, supporting sAXL evaluation in larger clinical studies and pointing to AXL as a potential target for HF therapy.

## 1. Introduction

Heart failure (HF) is a clinical syndrome caused by a deterioration of the heart’s function [1]. HF is a growing health concern linked to an aging population and the increasing prevalence of cardiovascular risk factors [2,3]. The most common HF causes include myocardial dysfunction due to coronary artery disease (CAD), hypertension, and valve disease, among other less prevalent causes. Its initial diagnosis is based on the presence of clinical symptoms and signs associated with cardiac dysfunction [1]. However, this lacks sufficient accuracy or specificity. Therefore, most clinical practice guidelines recommend the measurement of blood biomarkers such as natriuretic peptides to confirm HF diagnosis [4]. This area of research has been very active in recent years, as indicated by a large number of studies and reviews on this topic [5,6,7].

HF is classified into three main subtypes depending on left ventricular ejection fraction (EF). HF with reduced EF (HFrEF) is characterized by an EF ≤ 40%. However, roughly 50% of HF patients present an EF > 40%. This group of patients has been recently subdivided into two, those with preserved EF (≥50%; HFpEF) and an intermediate group named HF with mildly reduced EF (EF 41–49%; HFmrEF). The definition has important implications for the characteristics, prognosis, and treatment of HF. HFmrEF and HFrEF have a higher frequency of underlying CAD compared to those with HFpEF [8]. HFpEF is more frequently associated with hypertension and is more prevalent in women and older patients [9,10]. However, one should consider that EF is a continuous variable with a normal distribution in the population [11].

The use of cardiac markers with high prognostic value in HF evaluation is crucial for patient triage. Brain natriuretic peptide (BNP) and NT-proBNP, the products of the cleavage of pre-proBNP, have been the gold standard biomarkers in HF [12]. Elevated natriuretic peptide concentration associates with abnormal hemodynamics at the heart ventricle and cardiac dysfunction in HF. Employing the level of different natriuretic peptides in the diagnosis of acute HF is well established and included by guidelines in the clinical practice [1,13,14]. However, these guidelines mention that the use of other biomarkers apart from BNP or NT-proBNP should be considered for risk stratification in the management of HF [14]. The use of blood biomarkers is especially relevant in HFpEF, as natriuretic peptides are less elevated. In this context, we have proposed sAXL as a candidate to take on this role [15].

AXL is a receptor tyrosine kinase with functions in immune regulation and tissue homeostasis [16]. AXL is processed in the extracellular membrane of cells by ADAM10/17 proteolysis, releasing the extracellular portion of the molecule, known as soluble AXL (sAXL) [17]. Previous studies have shown that sAXL is increased in HF patients with reduced ejection fraction (HFrEF), correlating with an increased AXL abundance in cardiac tissue [15]. High sAXL levels are associated with a worse prognosis in HFrEF [15,18,19]. Furthermore, in patients suffering myocardial infarction with ST-segment elevation, sAXL levels are increased in patients undergoing adverse left ventricular remodeling [20].

These clinical studies suggested that AXL could influence multiple aspects of cardiovascular physiology via its diverse effects on vascular and immune cells [21]. Multiple studies suggest that after engaging its ligand GAS6, AXL drives vascular remodeling by regulating the biology of leukocytes, VSMCs, ECs, and pericytes, thereby facilitating pathological processes such as neointima proliferation in the vasculature induced by redox stress [22,23], flow [23] or mechanical injury [24,25,26]. More recently, several studies using animal models have shown that AXL influences on the response of the heart to damage. In rats subjected to thoracic transverse aortic constriction, *axl* expression increased, correlating with left ventricular hypertrophy. This rise was matched by the appearance of the soluble form of AXL in blood, which only increased in the initial hypertrophy group [27]. In a mouse strain where *axl* is depleted in myeloid cells, there is a reduction in proinflammatory cytokines after reperfusion in a myocardial infarction model [28], similar to what has been observed in livers subjected to profibrotic stimuli [29]. Inhibition of AXL while maintaining MerTK, a second GAS6 receptor, improves cardiac healing in those models [28]. AXL has also a prominent role in cardiac allograft vasculopathy. AXL-deficient recipient mice displayed fewer immune cells and reduced neointima formation in grafted vessels. This function was linked to AXL expression in myeloid cells [30]. Interestingly, *gas6* knockout animals also show an improved allograft heart survival, suggesting that GAS6 interaction with AXL is mediating this effect [31]. Indeed, the lack of GAS6 reduces leukocyte extravasation in different models of local inflammation [31]. All these studies employing different preclinical models suggest that AXL is a substantial player in heart physiology, especially in response to a chronic damage, as those involved in the development of HF.

An aspect that has not been evaluated yet in the literature is the specific role of AXL in HF with preserved ejection fraction. Therefore, here, we intend to study the prospect of sAXL as a prognostic biomarker in the context of HF. The specific objectives of the present study were (1) to define the value of sAXL as a biomarker in patients with chronic HFpEF patients compared to a similar cohort of HFrEF patients, (2) to compare its predictive performance of clinical outcomes with NT-proBNP measurements, and (3) to validate the prognostic sAXL value in HFrEF patients with a different cohort from our previous studies. First, we determined the serum sAXL concentration in a cohort of patients with HFpEF and compared the sAXL serum concentration with their concentrations in similar patients with HFrEF. Clinical outcomes were studied, including the endpoint of all-cause death (as the primary endpoint) and cardiovascular death or re-admission due to HF (as the secondary endpoint).

## 2. Materials and Methods

### 2.1. Study Design, Study Population and Ethics

Samples and data from patients included in this study were handled and provided by the Biobank HUB-ICO-IDIBELL (PT20/00171), integrated into the ISCIII Biobanks and Biomodels Platform. They were processed following standard operating procedures with the appropriate approval of the Ethics and Scientific Committees. The study population derives from DAMOCLES (Definition of the neuro-hormonal Activation, Myocardial function, genomic expression, and clinical OutComes in heart faiLurE patientS), an observational, prospective cohort study of 1236 consecutive chronic HF patients. The cohort was recruited between January 2004 and January 2013 at a single center. The methodology of the DAMOCLES study has been published previously [32,33]. Briefly, the inclusion criteria for the patients included consisted of a diagnosis of chronic HF following the European Society of Cardiology (ESC) criteria, to have had at least one recent acute decompensation of HF requiring intravenous diuretic therapy (either hospitalized or in the day-care hospital), and to be in stable condition at the time of inclusion in the study. Exclusion criteria were: significant primary valvular disease, clinical signs of fluid overload, pericardial disease, restrictive cardiomyopathy, hypertrophic cardiomyopathy, hemoglobin (Hb) concentration below 8.5 g/dL, chronic liver disease or active malignancy. The patients were recruited regardless of their percentage of left ventricular EF. The study was approved by the local ethics committee for clinical research and was conducted following the principles of the Declaration of Helsinki. All patients gave written informed consent before their inclusion in the DAMOCLES study.

### 2.2. Definition of Study Cohorts and Selection Criteria 

Using samples from the DAMOCLES study, we selected two different nested cohorts of patients with HF for the purpose of the present investigation. The two cohorts consisted of 134 HF patients from the DAMOCLES cohort study matching the criteria of HFrEF and HFpEF, respectively. A collection of 20 samples of unrelated, healthy blood donors from the same geographical area were used as a reference group.

### 2.3. Clinical Assessment at the Time of Inclusion

A baseline assessment was performed for all DAMOCLES participants at the study entry. This detailed evaluation included the collection of information about demographic characteristics, exhaustive medical history to gather clinical and disease-related factors: New York Heart Association (NYHA) functional class was recorded at enrollment in DAMOCLES based on patient symptoms, comorbidities, laboratory information, medical treatment, and the most recent determination of left ventricular EF (Table 1). The sources of information employed in order to generate the database of the study consisted in the patient’s medical history and standardized questionnaires.

### 2.4. Blood Sample Management 

Laboratory data and blood sample management methods have been previously reported by our group [32]. Blood samples were collected in serum tubes, immersed in ice, and immediately processed in aliquots of 250–500 µL. The resulting serum samples were frozen and stored at −80 °C using the Micronics^®^ (High Wycombe, UK) system. Samples and data from patients included in this study were provided by the Biobank HUB-ICO-IDIBELL (PT20/00171), integrated with the Spanish Biobank Network. Samples and data were processed following standard operating procedures with the appropriate approval of the Ethics and Scientific Committees.

### 2.5. Clinical Laboratory Determinations

Serum N-terminal pro b-type natriuretic peptide (NT-proBNP) concentration was measured in pg/mL using an immunoassay based on chemiluminescence with the Elecsys System (Roche^®^, Basel, Switzerland). This determination employs a two-step sandwich assay and was performed in Cobas^®^ analyzers. sAXL was measured in serum using a commercial sandwich ELISA, consisting of a capture monoclonal antibody recognizing the extracellular domain of AXL and a biotinylated polyclonal antibody linked to biotin for the detection step as previously described [34]. The ELISA was purchased from R and D systems and has been validated in [35]. Samples were diluted 1:50 in a solution containing 1% bovine serum albumin in phosphate-buffered saline (pH = 7.4). Hemoglobin levels in g/dL were obtained by laser-based impedance colorimetry. The glomerular filtration rate (GFR) was calculated from the determination of serum creatinine using the Modification of Diet in Renal Disease Study Group (MDRD) equation, a widely used parameter for measuring excretory kidney function [36]. Weight was recorded upon inclusion in order to estimate the body mass index (BMI) using the formula: BMI = weight (kg)/height (m^2^).

### 2.6. Follow-Up and Major Heart Failure Events Ascertainment

DAMOCLES study participants were followed for a median of 2.93 years (mean 3.3 years). Follow-up was conducted by trained study personnel and lasted until November 2015. The data on mortality and the cause of death were obtained from hospital and primary care electronic medical records, and/or by direct interview with the patients’ relatives.

### 2.7. Statistical Methods

All analyses were performed using the SPSS software (version 28.0; IBM, New York, NY, USA). Cross-sectional and longitudinal descriptive analyses were performed using the baseline and follow-up data from the DAMOCLES cohort [32,33]. Demographic characteristics, results from clinical laboratory tests and clinical characteristics, as well as laboratory tests results were summarized using basic descriptive statistics according to HF with reduced or preserved EF.

For categorical variables, number and percentage were reported, and for continuous variables median and interquartile range was used. χ2, Student’s T, and non-parametric tests were used to compare characteristics across strata.

The log-rank test was used to assess the association of each individual variable with survival. Survival curves were obtained using the Kaplan–Meier product limit estimator. The adjusted effect of important factors on patient survival was then determined with Cox proportional hazards regression using the forward stepwise method based on the likelihood ratio. Cox’s regression is a semi-parametric model widely used to establish association between predictors and time-to-event, as it makes fewer assumptions than parametric models. Two multivariable models were employed using age and NYHA class (model 1) and including NTproBNP and eGFR (model 2). The parameters included had clinical relevance in the etiology of HF (age; NYHA class; NTproBNP). eGFR was included in the multivariable model 2 as it has shown association with AXL in previous studies [21]. sAXL distribution values in quartiles were analyzed, and the 3rd quartile value was used as a cut-off point for stratification in the Kaplan–Meier survival curves. Subdivision in quartiles is useful analytical tool, as quantiles are less susceptible than means to long-tailed distributions and outliers. Patients were divided in two groups with sAXL below or equal the 3rd quartile (sAXL ≤ Q3) or above (>Q3). All statistical tests were solved fixing the probability of type I error (alpha) at 5%, and confidence intervals (CI) were obtained for a 95% likelihood. Values of *p* below 0.05 were considered statistically significant. 

## 3. Results

### 3.1. Characteristics of HF cohorts

The baseline characteristics of the HFrEF and HFpEF cohorts are shown in Table 1. Their demographic and clinical characteristics are consistent with those expected for each cohort. Patients with HFpEF had a higher proportion of women, were older and had a higher body mass index (BMI). Systolic blood pressure (SBP) was higher in the HFpEF, while the glomerular filtration rate (eGFR) was lower, indicative of a higher frequency of renal dysfunction in the HFpEF group. No differences were observed in diagnostic criteria for diabetes. Interestingly, while the proportion of NYHA III-IV patients was higher in the HFpEF group, there were no differences in all-cause mortality or major cardiovascular events during the follow-up, which were similar in both groups. Additionally, HFrEF had a higher ischemic etiology percentage, and more patients were treated with ACE/ARB and /or β-blockers compared to HFpEF patients.

### 3.2. sAXL Values Are Higher than a Group of Healthy Individuals and Similar in Both HF Groups

**Figure 1:**
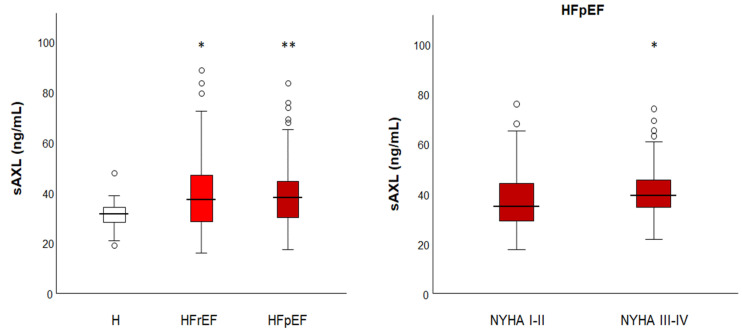
Box-plot representation of soluble AXL levels in serum by groups: healthy (H, white), HF with reduced ejection fraction (HFrEF, light red) and preserved ejection fraction (HFpEF, dark red) patients (left). Dots represent outliers. ANOVA analysis among groups was significant (*p* = 0.029). Post hoc analysis showed significant differences of HF groups vs. healthy individuals. Right, soluble AXL levels in HFpEF patients with lower NYHA class (I-II) compared to higher NYHA class (III-IV). Significance is denoted by * *p* < 0.05; ** *p* < 0.01.

Next, we measured the concentration of sAXL in serum of these HF samples. As there is no reference range established for sAXL in the general population, the results of the two groups were compared with a group of unrelated healthy individuals (n = 20) of the same geographical area (female 40%; age 61 [43–80]). The serum concentration of sAXL was higher in HFrEF patients, 37.2 ng/mL (IQR: 28.4–46.8; *p* = 0.004) and HFpEF patients, 37.9 ng/mL (IQR: 30.0–44.5; *p* < 0.001) compared to the healthy group, 31.5 ng/mL (IQR: 27.9–34.4; Figure 1). Both HF cohorts had similar sAXL levels (*p* = 0.807). HFpEF patients with NYHA class III–IV (n = 64) had also higher serum concentration of sAXL than those with NYHA class I–II (n = 70; *p* = 0.025, Figure 1).

### 3.3. Patient’s Prognosis According to sAXL and NTproBNP Levels in Serum

In an adjusted Cox proportional hazards model, serum sAXL concentration showed a significant association with all-cause mortality in both HF groups (Table 2). Interestingly, when cardiovascular death was considered, only the HFpEF group remained significant. No associations were observed for the end point of re-admission due to HF. Serum sAXL concentration was significantly associated with two combined end-points: major adverse event (re-admission due to HF or all-cause mortality), or major cardiovascular event (readmission or cardiovascular death) in the HFpEF group. In contrast, this was not the case in the HFrEF group, where sAXL was not significantly associated to re-admission due to HF or to cardiovascular mortality, nor with the combined end points. For comparison, we include in Table 2 the same analysis for NT-proBNP. The association with the different end points was much better for NT-proBNP compared to sAXL in the HFrEF group. However, in the HFpEF group, sAXL showed equal significance in the association with all-cause mortality and cardiovascular mortality than NT-proBNP.

We performed a multivariable regression analysis (Table 3), considering as predictors sAXL (ng/mL), age and NYHA class (model 1). Then, we included in the analysis NT-proBNP, and eGFR (model 2). Interestingly, sAXL was a predictor of time to death (all-cause) only in the HFpEF group, together with NT-proBNP, but not in the HFrEF group. In HFrEF, only age and NT-proBNP in model 2 remained significant. When only cardiovascular death was considered, the same pattern was observed, although in this case sAXL remained a better predictor than NT-proBNP, which did not reach significance. The multivariable model showed a similar result when additional parameters were added, including diabetes, hemoglobin, BMI, ischemic etiology, sex and number of comorbidities (results not shown). 

### 3.4. Patient’s Characteristics and Prognosis According to sAXL Levels in Serum

Next, we analyzed the characteristics and prognosis of both cohorts according to the baseline sAXL serum concentration divided in quartiles. HFrEF patients with sAXL > Q3 displayed higher concentration of NT-proBNP and hemoglobin, and a marked decrease in eGFR (Table 4). In this group, there was no difference in all-cause or cardiovascular-related mortality. HFpEF patients in the highest sAXL quartile had increased all-cause and cardiovascular mortality, compared to those with lower sAXL. No differences were observed in age, SBP, eGFR, BMI. Interestingly, patients with sAXL > Q3 had only marginally higher NT-proBNP, and a very significant decrease in hemoglobin (Table 4).

**Figure 2:**
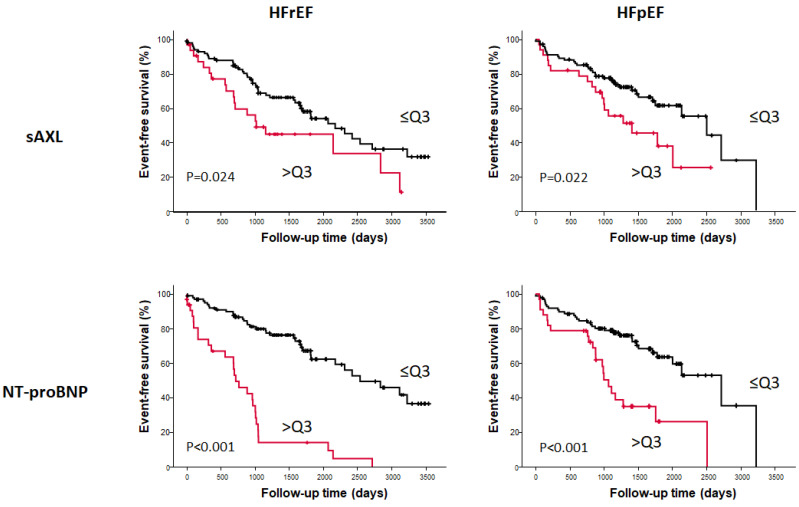
Kaplan–Meier survival curves from HF patients divided according their sAXL levels (**upper** panels) and NT-proBNP levels (**lower** panels). The graphs represent the survival from all-cause mortality. Left panels, Kaplan–Meier event-free survival curve from HF patients with reduced ejection fraction (HFrEF) and right panels, Kaplan–Meier event-free survival curve from HF patients with preserved ejection fraction (HFpEF). Survival of patients with sAXL or NT-proBNP values below the 4th quartile cut-off point is shown in black line, while survival of patients with sAXL or NT-proBNP above the 3rd quartile is shown in red line. The differences in survival were compared with the Cox Regression model.

In a survival analysis, HFrEF patients with sAXL values in the highest quartile (>Q3) had a poorer outcome, with a median survival of 1019 days (IQR: 573–1464), compared to those with lower sAXL (2176 days, IQR: 1616-2735; log rank *p* = 0.024). This was also observed in HFpEF patients, where sAXL > Q3 had a mean survival of 1408 days (IQR: 626–2190) compared to 2506 days (IQR: 1741–3271) in those ≤Q3 (log rank *p* = 0.022). The survival curves are shown in Figure 2. Similarly, a Cox regression univariable model, using the lowest quartile as baseline, showed that HFpEF patients with sAXL in > Q3 had a significant association with all-cause mortality (HR of 3.6), while the association was not significant in the HFrEF group (Table 5). Similar results were obtained for cardiovascular mortality.

In order to evaluate the performance of serum sAXL as a valuable HF biomarker in each group of patients, we analyzed the predictive value of NT-proBNP dividing the cohorts in quartiles and compared the result with the performance of sAXL (Figure 2). As expected, the natriuretic peptide was a very good marker of the severity of the disease in both groups (Figure 2), as reflected in the HFrEF by a median survival time in the >Q3 group of 716 days (IQR: 463–969), compared with 2541 days (IQR: 1711-3371) for the rest of patients in the HFrEF cohort (log rank *p* < 0.0001). In the HFrEF cohort, NT-proBNP >Q3 was equally a very good predictor of mean survival: days 1066 (IQR:906–1226) compared to 2718 days (IQR: 1825–3612) for those in ≤Q3 (log rank *p* < 0.0001). 

## 4. Discussion

Studies using preclinical models have shown that the AXL receptor tyrosine kinase could be considered a potential target in cardiac diseases [21]. In particular, several reports in the literature have suggested that modulating specifically the GAS6/AXL interaction would improve chronic heart failure [21]. Studies of patients suffering from this condition have also pointed to the role of GAS6/AXL in the pathological processes of a deteriorating heart. End-stage HF patients undergoing transplantation had increased AXL in the heart, while a group of chronic HFrEF patients had sAXL concentration in serum 25% higher than healthy individuals [15]. Here, we observed a 23% increase in the mean concentration of the HFrEF group from the DAMOCLES cohort (Table 1). Further, we extended this observation to an HFpEF group, finding a quantitatively similar elevation of the serum sAXL concentration (26%) compared to a group of unrelated healthy individuals. In patients that develop HF after a myocardial infarction (MI) with Killip>1, this increase in sAXL seems to occur progressively. Interestingly, there was an association of sAXL at seven days post MI with left ventricular remodeling [20], indicative of an activation of AXL signaling or its processing in the initial stages of heart failure in connection with cardiac structural changes in the initial stages of heart failure. In the context of heart transplantation, increased sAXL after transplant could also reflect the fitness of the organ, as its levels are higher in patients having a cardiovascular event during a three-year follow-up [37].

The concentration of sAXL in blood seems to be a valuable biomarker of prognosis in patients with chronic HF. In this respect, the prognosis of HFrEF after one-year follow up was worse in the group of patients with sAXL concentration in the highest quartile [15]. This was confirmed in a prolonged follow-up (3.6 years) in the same group of patients [18]. Here we could confirm that the association of high sAXL concentration and worse prognosis was not restricted to reduced EF; HFpEF patients also displayed increased all-cause mortality if the sAXL was in the highest quartile. In a multivariable analysis, the difference was confirmed, and only the NT-proBNP and sAXL values were independent risk factors for all-cause mortality. Clearly, in both groups, NT-proBNP was a very good prognostic marker of survival, as has been already demonstrated in other studies [38,39,40]. As mentioned by Salah et al. (2019), comorbidities could contribute relatively more to prognosis in patients with HFpEF with lower NT-proBNP levels than in patients with HFrEF. In this context, the determination of sAXL when a person is diagnosed with HF could provide additional information to NT-proBNP measurements [39]. In a recent analysis of the DRAGON-HF trial, Liu and coworkers have confirmed that sAXL could predict clinical outcomes in a large population (>1000 symptomatic HF patients) independent of NT-proBNP [19]. Interestingly, when only cardiovascular mortality was considered, sAXL increased its association with survival [19]. In our study, this was found specifically in the HFpEF group. In HFpEF, NT-proBNP was a worse predictor than sAXL for cardiovascular mortality (Table 2).

The fact that NT-proBNP and sAXL have different diagnostic and prognostic values for HFrEF and HFpEF emphasizes the divergent etiological causes of both HF entities. HFpEF is becoming the most common HF form due to the ageing of the population and the increase in the prevalence of obesity, metabolic syndrome, and diabetes mellitus [3,9]. Despite this, many clinical trials, including effective HFrEF medication, have not succeeded to identify treatments for HFpEF. A putative explanation for such failure could be that patients with HFpEF have different pathophysiological pathways activated and therefore, they should be treated differently [9]. Further research on whether sAXL could identify a subset of HFpEF patients with a common phenotype is warranted with larger patient cohorts. Our results indicate that sAXL is elevated in HFpEF patients and due to the implication of AXL in many cardiac pathologies, as described, it could be a determinant factor in HFpEF development. Comparing sAXL with other suggested biomarkers in HF would be informative, including ST2 and troponins. ST2 is a fragment of the interleukin 1 receptor-like 1 [41,42,43]. Therefore, this molecule shares similarities with sAXL, as both molecules originate by receptor shedding in the cellular membrane and are implicated in immunomodulation. Troponin concentration is prognostic in acute and chronic heart failure and has been suggested as a tool for a personalized approach to HF management [44,45]. Previously, we compared the performance of sAXL with troponin T in a chronic HFrEF cohort and in heart transplant patients, showing that both biomarkers are independent predictors of adverse outcomes [18,37]. Further studies using these biomarkers are necessary to establish their relative clinical utility in HF.

There are limitations in our analysis, including that the comparison of HF groups was made to a reference group that was not related to the HF cohorts and had different demographic characteristics. Further, as the inclusion and follow-up finished in 2015, more recent groups of HF patients should be considered in order to include the most recent therapies used in HF. The effect of these therapeutic regimes on the plasma biomarkers should be studied. The inclusion of the patients in the preserved or reduced EF groups was performed in order to maximize the different characteristics of each group while avoiding patients that could be considered in the intermediate group. In this sense, our groups represent those patients with a clear diagnosis of HFpEF and HFrEF. This selection could explain the reduced association of sAXL with mortality in the HFrEF observed in the present study compared to previous ones with different inclusion criteria [15,18,19]. In addition, one should be cautious in considering the role of AXL in the context of HF. AXL activation might represent a restorative signal in the heart, with increased sAXL levels reflecting the activation of the pathway in the context of cardiac damage. At present, it is still necessary to acquire a better knowledge of the complex role of the GAS6/AXL system in cardiovascular biology.

## 5. Conclusions

To sum up, in our study, serum concentration of sAXL is shown to be elevated in both HFpEF and HFrEF groups, providing a significant measure for determining the prognosis of the disease. The consistent increase in sAXL associated with HF could reflect an increased activation of the GAS6/AXL system, paralleling the evolution of the disease. AXL is already a valuable target in other pathologies [46,47], and several drugs have been developed to regulate its activity specifically. Our results reveal sAXL as a predictor of time to death (all-cause) in HFpEF patients. Of note, sAXL has a similar predictive association to NT-proBNP in the HFpEF cohort, justifying its future evaluation in larger studies and pointing to AXL as a promising target for HF therapy.

## Figures and Tables

**Table 1:** Baseline characteristics of heart failure patients with reduced (HFrEF) and preserved (HFpEF) ejection fraction.

	HFrEF	HFpEF	*p*
Number	134	134	
Demographics			
Age (years)	71 (61–78)	78 (71–82)	<0.001
Female, n (%)	41 (30.6)	87 (64.9)	<0.001
Risk Factors, n (%)			
Diabetes, n (%)	63 (47.0)	73 (54.5)	0.222
Ischemic etiology, n (%)	68 (50.7)	26 (19.4)	<0.001
Clinical characteristics and outcomes			
BMI (kg/m^2^)	27.3 (24.4–30.8)	29.5 (26.3–33.3)	0.001
NYHA FC I-II / III-IV, n (%)	89 (66.4)/45 (33.6)	70 (52.2)/64 (47.8)	0.018
EF	32 (31–33)	62 (60–64)	<0.001
SBP (mmHg)	120 (107–130)	129 (115–141)	<0.001
Comorbodities number > 4, n (%)	59 (44.4)	69 (53.4)	0.088
All-cause mortality	64 (47.8)	53 (39.6)	0.175
Cardiovascular mortality	20 (14.9)	20 (14.9)	1.000
Re-admission	49 (36.6)	42 (31.3)	0.367
Re-admission or exitus (all-cause)	80 (59.7)	75 (56.0)	0.536
Re-admission or exitus (CV)	54 (40.3)	51 (38.1)	0.707
Laboratory variables			
eGFR	60.6 (47.0–76.3)	52.5 (38.0–68.7)	0.009
sAXL (ng/mL)	37.2 (28.4–46.8)	37.9 (30.0–44.5)	0.612
NT-proBNP	3.22 (2.78–3.54)	3.06 (2.78–3.43)	0.061
Medications (%)			
ACE/ARB, n (%)	111 (82.8)	90 (67.2)	0.003
β-blocker, n (%)	128 (95.5)	109 (81.3)	<0.001
Hydralazine, n (%)	23 (17.2)	31 (23.1)	0.235

Data are presented as median (IQR) or n (%). The *p*-value is calculated for continuous parameters with the Mann–Whitney U test, and for categorical parameters with the chi-square test or exact Fisher text; *p* < 0.05 is considered significant. BMI, body mass index; NYHA FC New York Heart Association functional class; EF ejection fraction; SBP, systolic blood pressure; eGFR, Creatinine clearance measured as MDRD; sAXL soluble AXL; NT-proBNP, N-terminal pro brain natriuretic peptide; ACE, angiotensin converting enzyme inhibitor; ARB, angiotensin receptor blocker.

**Table 2:** Cox regression value for absolute sAXL.

	HFrEF		HFpEF	
	HR (95% CI)	*p*	HR (95% CI)	*p*
All-cause mortality				
sAXL	1.019 (1.000–1.038)	0.047	1.032 (1.013–1.052)	<0.001
NT-proBNP	1.050 (1.036–1.066)	<0.001	1.021 (1.021–1.050)	<0.001
Cardiovascular mortality				
sAXL	0.991 (0.954–1.030)	0.647	1.054 (1.025–1.083)	<0.001
NT-proBNP	1.065 (1.040–1.091)	<0.001	1.041 (1.020–1.063)	<0.001
Re-admission				
sAXL	1.000 (0.977–1.024)	0.995	1.002 (0.977–1.027)	0.904
NT-proBNP	1.037 (1.019–1.055)	<0.001	1.025 (1.006–1.044)	0.011
Re-admission or exitus (all-cause)				
sAXL	1.010 (0.993–1.027)	0.27	1.020 (1.003–1.037)	0.020
NT-proBNP	1.042 (1.028–1.055)	<0.001	1.030 (1.016–1.045)	<0.001
Re-admission or exitus (Cardiovascular)				
sAXL	1.002 (0.981–1.024)	0.832	1.022 (1.002–1.042)	0.028
NT-proBNP	1.041 (1.024–1.058)	<0.001	1.032 (1.015–1.048)	<0.001

Hazard ratios (HR) of sAXL or NT-proBNP for clinical end-points in heart failure (HF) patients. Patients are divided as HF with reduced ejection fraction (HFrEF) or preserved ejection fraction (HFpEF). Data is represented as HR (95% CI; confidence intervals). The *p* value is calculated with Cox proportional hazards models; *p* < 0.05 is considered significant.

**Table 3:** Multivariable Cox regression value for absolute sAXL.

	HFrEF				HFpEF			
	**Model 1**		**Model 2**		**Model 1**		**Model 2**	
	HR (95% CI)	*p*	HR (95% CI)	*p*	HR (95% CI)	*p*	HR (95% CI)	*p*
**All-cause mortality**								
sAXL	1.018 (0.998–1.037)	0.073	1.005 (0.982–1.028)	0.693	1.028 (1.008–1.048)	0.005	1.024 (1.002–1.046)	0.033
Age	1.069 (1.041–1.098)	<0.001	1.042 (1.046–1.076)	0.002	1.024 (0.989–1.061)	0.186	1.030 (0.868–2.959)	0.132
NYHA class	1.463 (0.877–2.441)	0.145	1.238 (0.737–2.079)	0.420	1.805 (0.993–3.280)	0.053	1.603 (0.868–2.959)	0.132
NTproBNP			1.037 (1.020–1.055)	<0.001			1.026 (1.009–1.044)	0.003
eGFR			0.999 (0.988–1.010)	0.788			0.989 (0.974–1.005)	0.182
**Cardiovascular mortality**								
sAXL	0.989 (0.951–1.029)	0.586	0.952 (0.907–1.000)	0.045	1.051 (1.022–1.081)	<0.001	1.049 (1.018–1.081)	0.002
Age	1.066 (1.018–1.115)	0.006	1.023 (0.978–1.070)	0.264	1.054 (0.988–1.124)	0.103	1.077 (1.007–1.153)	0.030
NYHA class	1.625 (0.663–3.982)	0.288	1.520 (0.596–3.877)	0.429	1.706 (0.632–1.124)	0.292	1.522 (0.551–4.209)	0.418
NTproBNP			11.786 (3.61–38.46)	<0.001			1.034 (1.007–1.062)	0.013
eGFR			1.004 (0.989–1.019)	0.472			1.003 (0.978–1.028)	0.833

Multivariable Cox regression analysis for sAXL; age and NYHA class (model 1) with addition of NT-proBNP; N-terminal pro brain natriuretic peptide and eGFR: estimated Glomerular Filtration Rate (model 2) with all-cause and cardiovascular mortality end-points. Patients are divided as HF with reduced ejection fraction (HFrEF) or preserved ejection fraction (HFpEF). Data is represented as HR (95% CI; confidence intervals). The P value is calculated with Cox proportional regression model using Forward Stepwise Likelihood Ratio Method; *p* < 0.05 is considered significant.

**Table 4:** Heart failure patients’ characteristics according to the sAXL quartile.

	HFrEF			HFpEF		
	sAXL ≤ Q3	sAXL > Q3	P	sAXL ≤ Q3	sAXL > Q3	P
Number	101	33		101	33	
AC mortality n (%)	45 (44.5)	19 (57.6)	0.136	35 (34.6)	18 (54.5)	0.035
CV mortality n (%)	3 (9.01)	17 (16.8)	0.279	11 (10.9)	9 (27.3)	0.022
NYHA III-IV	32 (31.7)	13 (39.4)	0.271	47 (46.5)	17 (51.5)	0.383
SBP (mm Hg)	120 (106–130)	120 (110-131)	0.329	129 (115-140)	129 (113-147)	0.616
Re-ad. or exitus n (%)	40 (39.6)	14 (42.4)	0.465	34 (33.7)	17 (51.5)	0.053
Sex (male) n (%)	72 (71.3)	21 (63.6)	0.268	38 (37.6)	9 (27.3)	0.193
Diabetes n (%)	45 (44.5)	18 (54.5)	0.213	54 (53.5)	19 (57.6)	0.418
eGFR	62.2 (52.5–78.2)	42.1 (23.9–66.2)	<0.001	52.5 (38.5)	52.4 (34.8–69.2)	0.784
BMI	27.3 (24.1–30.8)	27.3 (24.9–30.7)	0.749	29.6 (26.3–33.0)	27.8 (25.4–35.6)	0.848
Age (years)	69 (60.0–78.0)	75 (67.5–78.5)	0.069	78.0 (71.0–83.0)	77.5 (71.5–79.5)	0.776
NT-proBNP	3.13 (2.73–3.49)	3.49 (3.15–3.96)	<0.001	3.02 (2.76–3.33)	3.28 (2.84–3.63)	0.050
Hemoglobin (g/dL)	13.1 (12.3–14.2)	12.4 (10.8–13.4)	0.005	12.2 (11.05–14)	11.1 (10.0–11.9)	<0.001

**Table 5:** Cox regression for absolute sAXL levels in quartiles and all cause-mortality.

	HFrEF	HFpEF
**Q1**	**1**	**1**
**Q2**	0.621 (0.291–1.327)	2.222 (0.883–5.592)
**Q3**	0.947 (0.466–1.923)	1.908 (0.776–4.690)
**Q4**	1.584 (0.837–2.988)	3.628 (1.499–8.782)

## Data Availability

Individual de-identified data are available from the authors upon request.

## References

[1] McDonagh T.A., Metra M., Adamo M., Gardner R.S., Baumbach A., Böhm M., Burri H., Butler J., Čelutkienė J., Chioncel O. (2021). 2021 ESC Guidelines for the diagnosis and treatment of acute and chronic heart failure. Eur. Heart J..

[2] Farré N., Vela E., Clèries M., Bustins M., Cainzos-Achirica M., Enjuanes C., Moliner P., Ruiz S., Verdú-Rotellar J.M., Comín-Colet J. (2017). Real world heart failure epidemiology and outcome: A population-based analysis of 88,195 patients. PLoS ONE.

[3] Groenewegen A., Rutten F.H., Mosterd A., Hoes A.W. (2020). Epidemiology of heart failure. Eur. J. Heart Fail..

[4] Mueller C., McDonald K., de Boer R.A., Maisel A., Cleland J.G., Kozhuharov N., Coats A.J., Metra M., Mebazaa A., Ruschitzka F. (2019). Heart Failure Association of the European Society of Cardiology practical guidance on the use of natriuretic peptide concentrations. Eur. J. Heart Fail..

[5] Berezin A.E., Berezin A.A. (2023). Biomarkers in Heart Failure: From Research to Clinical Practice. Ann. Lab. Medicine.

[6] Gui X.Y., Rabkin S.W. (2022). C-Reactive Protein, Interleukin-6, Trimethylamine-N-Oxide, Syndecan-1, Nitric Oxide, and Tumor Necrosis Factor Receptor-1 in Heart Failure with Preserved Versus Reduced Ejection Fraction: A Meta-Analysis. Curr. Heart Fail. Rep..

[7] Eltelbany M., Shah P., deFilippi C. (2022). Biomarkers in HFpEF for Diagnosis, Prognosis, and Biological Phenotyping. Curr. Heart Fail. Rep..

[8] Vedin O., Lam C.S., Koh A.S., Benson L., Teng T.H.K., Tay W.T., Braun O.Ö., Savarese G., Dahlström U., Lund L.H. (2017). Significance of ischemic heart disease in patients with heart failure and preserved, midrange, and Reduced Ejection Fraction: A Nationwide Cohort Study. Circ. Heart Fail..

[9] Borlaug B.A. (2020). Evaluation and management of heart failure with preserved ejection fraction. Nat. Rev. Cardiol..

[10] Lam C.S., Arnott C., Beale A.L., Chandramouli C., Hilfiker-Kleiner D., Kaye D.M., Ky B., Santema B.T., Sliwa K., Voors A.A. (2019). Sex differences in heart failure. Eur. Heart J..

[11] Stewart S., Playford D., Scalia G.M., Currie P., Celermajer D.S., Prior D., Codde J., Strange G., NEDA Investigators (2021). Ejection fraction and mortality: A nationwide register-based cohort study of 499 153 women and men. Eur. J. Heart Fail..

[12] Kuwahara K. (2021). The natriuretic peptide system in heart failure: Diagnostic and therapeutic implications. Pharmacol. Ther..

[13] Roberts E., Ludman A.J., Dworzynski K., Al-Mohammad A., Cowie M.R., McMurray J.J., Mant J. (2015). The diagnostic accuracy of the natriuretic peptides in heart failure: Systematic review and diagnostic meta-analysis in the acute care setting. BMJ.

[14] McDonagh T.A., Metra M., Adamo M., Gardner R.S., Baumbach A., Böhm M., Burri H., Butler J., Čelutkienė J., Chioncel O. (2022). 2021 ESC Guidelines for the diagnosis and treatment of acute and chronic heart failure. Eur. Heart.

[15] Batlle M., Recarte-Pelz P., Roig E., Castel M.A., Cardona M., Farrero M., Ortiz J.T., Campos B., Pulgarín M.J., Ramírez J. (2014). AXL receptor tyrosine kinase is increased in patients with heart failure. Int. J. Cardiol..

[16] Ranta A., Kumar S. (2020). Recent advancements in role of TAM receptors on efferocytosis, viral infection, autoimmunity, and tissue repair. Int. Rev. Cell Mol. Biol..

[17] Miller M.A., Sullivan R.J., Lauffenburger D.A. (2017). Molecular pathways: Receptor ectodomain shedding in treatment, resistance, and monitoring of cancer. Clin. Cancer Res..

[18] Batlle M., Campos B., Farrero M., Cardona M., González B., Castel M.A., Ortiz J., Roig E., Pulgarín M.J., Ramírez J. (2016). Use of serum levels of high sensitivity troponin T, galectin-3 and C-terminal propeptide of type I procollagen at long term follow-up in heart failure patients with reduced ejection fraction: Comparison with soluble AXL and BNP. Int. J. Cardiol..

[19] Liu Y., Wang X., Pan X., Ma T., Xu Y., Fen Q., Nijiati M., Chi C., Su Y., Zhang X. (2022). Prognostic value of plasma sAXL in patients with heart failure: Insights from the DRAGON-HF trial. ESC Heart Fail..

[20] Caldentey G., García De Frutos P., Cristóbal H., Garabito M., Berruezo A., Bosch X., San Antonio R., Flores-Umanzor E., Perea R.J., De Caralt T.M. (2019). Serum levels of Growth Arrest-Specific 6 protein and soluble AXL in patients with ST-segment elevation myocardial infarction. Eur. Heart J. Acute Cardiovasc. Care.

[21] McShane L., Tabas I., Lemke G., Kurowska-Stolarska M., Maffia P. (2019). TAM receptors in cardiovascular disease. Cardiovasc. Res..

[22] Konishi A., Aizawa T., Mohan A., Korshunov V.A., Berk B.C. (2004). Hydrogen peroxide activates the Gas6-Axl pathway in vascular smooth muscle cells. J. Biol. Chem..

[23] Liang Z., Yang Y., Wu X., Lu C., Zhao H., Chen K., Zhao A., Li X., Xu J. (2022). GAS6/Axl is associated with AMPK activation and attenuates H_2_O_2_-induced oxidative stress. Apoptosis.

[24] Korshunov V.A., Berk B.C. (2003). Flow-Induced Vascular Remodeling in the Mouse: A Model for Carotid Intima-Media Thickening. Arter. Thromb Vasc. Biol..

[25] Melaragno M.G., Wuthrich D.A., Poppa V., Gill D., Lindner V., Berk B.C., Corson M.A. (1998). Increased expression of Axl tyrosine kinase after vascular injury and regulation by G protein-coupled receptor agonists in rats. Circ. Res..

[26] Chen W., Van Beusecum J.P., Xiao L., Patrick D.M., Ao M., Zhao S., Lopez M.G., Billings F.T., Cavinato C., Caulk A.W. (2022). Role of Axl in target organ inflammation and damage due to hypertensive aortic remodeling. Am. J. Physiol. Heart Circ. Physiol..

[27] Batlle M., Castillo N., Alcarraz A., Sarvari S., Sangüesa G., Cristóbal H., García de Frutos P., Sitges M., Mont L., Guasch E. (2019). Axl expression is increased in early stages of left ventricular remodeling in an animal model with pressure-overload. PLoS ONE.

[28] DeBerge M., Glinton K., Subramanian M., Wilsbacher L.D., Rothlin C.V., Tabas I., Thorp E.B. (2021). Macrophage AXL receptor tyrosine kinase inflames the heart after reperfused myocardial infarction. J. Clin. Investig..

[29] Tutusaus A., de Gregorio E., Cucarull B., Cristóbal H., Aresté C., Graupera I., Coll M., Colell A., Gausdal G., Lorens J.B. (2020). A Functional Role of GAS6/TAM in Nonalcoholic Steatohepatitis Progression Implicates AXL as Therapeutic Target. Cell Mol. Gastroenterol. Hepatol..

[30] Glinton K., DeBerge M., Fisher E., Schroth S., Sinha A., Wang J.J., Wasserstrom J.A., Ansari M.J., Zhang Z.J., Feinstein M. (2021). Bone marrow-derived AXL tyrosine kinase promotes mitogenic crosstalk and cardiac allograft vasculopathy. J. Heart Lung Transpl..

[31] Tjwa M., Bellido-Martin L., Lin Y., Lutgens E., Plaisance S., Bono F., Delesque-Touchard N., Hervé C., Moura R., Billiau A.D. (2008). Gas6 promotes inflammation by enhancing interactions between endothelial cells, platelets, and leukocytes. Blood.

[32] Gavaldà-Manso M., Jimenez-Marrero S., Cainzos-Achirica M., Garay A., Enjuanes C., Yun S., Diez C., Gonzalez-Costello J., Tajes M., Farre N. (2019). Reduced levels of vasopressin, an independent mechanism in the obesity paradox in patients with chronic heart failure: Insights from the DAMOCLES study. Int. J. Cardiol..

[33] Díez-López C., Tajes Orduña M., Enjuanes Grau C., Moliner Borja P., González-Costello J., García-Romero E., Francesch Manzano J., Yun Viladomat S., Jiménez-Marrero S., Ramos-Polo R. (2021). Blood Differential Gene Expression in Patients with Chronic Heart Failure and Systemic Iron Deficiency: Pathways Involved in Pathophysiology and Impact on Clinical Outcomes. J. Clin. Med..

[34] Martínez-Bosch N., Cristóbal H., Iglesias M., Gironella M., Barranco L., Visa L., Calafato D., Jiménez-Parrado S., Earl J., Carrato A. (2022). Soluble AXL is a novel blood marker for early detection of pancreatic ductal adenocarcinoma and differential diagnosis from chronic pancreatitis. EBioMedicine.

[35] Dengler M., Huber H., Müller C.J., Zellmer A., Rauch P., Mikulits W. (2016). Accurate Determination of Soluble Axl by Enzyme-Linked Immunosorbent Assay. Assay Drug Dev. Technol..

[36] Gama R.M., Peracha J., Bramham K., Cockwell P. (2023). Removal of ethnicity adjustment for creatinine-based estimated glomerular filtration rate equations. Ann Clin Biochem..

[37] Mirabet S., García-Osuna A., Garcia de Frutos P., Ferrero-Gregori A., Brossa V., Lopez L., Leta R., Garcia-Picart J., Padro J.M., Sánchez-Quesada J.L. (2018). High-Sensitivity Troponin T and Soluble Form of AXL as Long-Term Prognostic Biomarkers after Heart Transplantation. Dis Markers.

[38] Santema B.T., Kloosterman M., Van Gelder I.C., Mordi I., Lang C.C., Lam C.S., Anker S.D., Cleland J.G., Dickstein K., Filippatos G. (2018). Comparing biomarker profiles of patients with heart failure: Atrial fibrillation vs. sinus rhythm and reduced vs. preserved ejection fraction. Eur. Heart J..

[39] Salah K., Stienen S., Pinto Y.M., Eurlings L.W., Metra M., Bayes-Genis A., Verdiani V., Tijssen J.G., Kok W.E. (2019). Prognosis and NT-proBNP in heart failure patients with preserved versus reduced ejection fraction. Heart.

[40] Lam C.S.P., Gamble G.D., Ling L.H., Sim D., Leong K.T.G., Yeo P.S.D., Ong H.Y., Jaufeerally F., Ng T.P., Cameron V.A. (2018). Mortality associated with heart failure with preserved vs. reduced ejection fraction in a prospective international multi-ethnic cohort study. Eur. Heart J..

[41] Torrente-Rodríguez R.M., Martín C.M., Gamella M., Pedrero M., Martínez-Bosch N., Navarro P., García de Frutos P., Pingarrón J.M., Campuzano S. (2021). Electrochemical Immunosensing of ST2: A Checkpoint Target in Cancer Diseases. Biosensors.

[42] Emdin M., Aimo A., Vergaro G., Bayes-Genis A., Lupón J., Latini R., Meessen J., Anand I.S., Cohn J.N., Gravning J. (2018). sST2 Predicts Outcome in Chronic Heart Failure Beyond NT-proBNP and High-Sensitivity troponin T. J. Am. Coll. Cardiol..

[43] Ponikowska B., Iwanek G., Zdanowicz A., Urban S., Zymliński R., Ponikowski P., Biegus J. (2022). Biomarkers of Myocardial Injury and Remodeling in Heart Failure. J. Pers. Med..

[44] Agdashian D., Daniels L.B. (2023). What Is the Clinical Utility of Cardiac Troponins in Heart Failure? Are They Modifiable Beyond Their Prognostic Value?. Curr. Heart Fail. Rep..

[45] Aimo A., Januzzi J.L., Vergaro G., Ripoli A., Latini R., Masson S., Magnoli M., Anand I.S., Cohn J.N., Tavazzi L. (2018). Prognostic Value of High-Sensitivity Troponin T in Chronic Heart Failure: An Individual Patient Data Meta-Analysis. Circulation.

[46] Tutusaus A., Marí M., Ortiz-Pérez J.T., Nicolaes G.A.F., Morales A., García de Frutos P. (2020). Role of Vitamin K-Dependent Factors Protein S and GAS6 and TAM Receptors in SARS-CoV-2 Infection and COVID-19-Associated Immunothrombosis. Cells.

[47] Bárcena C., Stefanovic M., Tutusaus A., Joannas L., Menéndez A., García-Ruiz C., Sancho-Bru P., Marí M., Caballeria J., Rothlin C.V. (2015). Gas6/Axl pathway is activated in chronic liver disease and its targeting reduces fibrosis via hepatic stellate cell inactivation. J. Hepatol..

